# Prevalence of Orthodontic Treatment Need and Occlusal Traits in Schoolchildren

**DOI:** 10.1155/2014/349793

**Published:** 2014-10-28

**Authors:** Abdolreza Jamilian, Alireza Darnahal, Elnaz Damani, Maziar Talaeipour, Zinat Kamali

**Affiliations:** ^1^Fellow of Orthognathic Surgery, Department of Orthodontics, Center of Craniofacial Research, Islamic Azad University, Dental Branch, No. 2713, Vali Asr Street, Tehran 1966843133, Iran; ^2^Department of Orthodontics, Islamic Azad University, Dental Branch, Tehran 1946853314, Iran; ^3^Department of Periodontics, Islamic Azad University, Dental Branch, Tehran 1946853314, Iran; ^4^National Nutrition and Food Technology Research Institute, Faculty of Nutrition Sciences and Food Technology, Shahid Beheshti University of Medical Sciences, Tehran 1985717443, Iran

## Abstract

*Background*. Widespread use of the IOTN along with detailed study of occlusal traits is suitable for planning community dental health resources. *Objectives*. The aim of current study was to assess the need for orthodontic treatment among school children of Tehran by means of the Dental Health Component (DHC) of the Index of Orthodontic Treatment Need (IOTN) and also to evaluate the occlusal traits of the subjects. *Methods*. 684 (343 boys and 341 girls) school children, 15 to 17 years of age, were selected at random from 12 schools to represent the four main areas of Tehran. The final sample who met the inclusion criteria comprised 643 subjects (322 males and 321 females). Malocclusion was determined with the Index of Orthodontic Treatment Need. The IOTN grades were statistically compared in the two genders using chi-square test. *Results*. Orthodontic treatment need, using the DHC, was found in only 9.0 per cent of the children. The prevalence of Angle Class I malocclusion in this study was higher than other malocclusions (65.2 per cent), followed by crowding in 62.7 per cent of the subjects. *Conclusion*. Orthodontic treatment need for Tehran high school students was relatively lower than that reported in most recent studies in Europe.

## 1. Introduction

Malocclusion in itself is neither a disease nor a life threatening condition [1]; nevertheless, the appearance of the mouth and smile plays a significant role in judgments regarding facial attractiveness. Thus, malocclusion has large physical, social, and psychological impact on the individual and society [2, 3]. Several studies have attempted to provide epidemiological reports of the prevalence of malocclusions in different ethnic groups [4–6]. In the last four decades scientists have proposed several indices for scoring how much the teeth deviate from the normal, as indicators of orthodontic treatment need. Grainger's Treatment Priority Index (TPI) [7] which was proposed in 1960s can be named as one of the most prominent ones since it was widely used in the 1965–1970 US population surveys. Recently, the Index of Treatment Need (IOTN) was proposed by Brook and Shaw [8] in the United Kingdom as a scoring system for estimating treatment need of patients with various degrees of malocclusion. This index places patients in five grades from “no need for treatment” to “extreme treatment need” (Table 1).

A few investigations have evaluated the prevalence of malocclusion in Tehran, but none of these investigations studied the details of malocclusions. Therefore, the purpose of the present epidemiologic study was to evaluate the orthodontic variables of 15–17 year-old schoolchildren in Tehran by means of the IOTN.

## 2. Material and Methods

This study received approval from the Human Research Ethics Committee of IAU of Medical Sciences. Twelve schools were selected among all high schools in east, west, north, south, and center of Tehran (Iran) by table of random numbers and 684 of the children attending these schools were also selected by table of random numbers. The study subject included 343 boys and 341 girls with the age range of 15–17 years. Parents of sampled children were notified about purposes of the study and informed written consent was obtained from each study participant and their parents. This study has been approved by the appropriate ethics committee and has therefore been performed in accordance with the ethical standards laid down in the 1964 Declaration of Helsinki and its later amendments.

Each patient was examined for orthodontic treatment need with Dental Health Component (DHC) of the Index of Orthodontic Treatment Need (IOTN). The examination lasted approximately 15 minutes per child, following the World Health Organization guidelines [9]. The clinical examination was carried out by a dentist who had previously undergone calibration by two orthodontists to standardize his procedures. The examinations were conducted at school during daylight with the help of tongue blade, mirror, probe, and ruler and no radiographs were taken. Treatment needs of the patients were categorized as grade 1 (no treatment need), grade 2 (mild need), grade 3 (moderate need), grade 4 (severe need), and grade 5 (extreme need) (Table 1). The subjects with previous orthodontic treatment were also categorized as a separate group. Orthodontic variables of the subjects including molar relationships, clinically absent teeth, overjet, overbite, open bite, deep bite, crossbite, scissor bite, crowding, lip competency, diastema, syndromes, and temporomandibular joint disorders including clicking, popping, or grating sounds in the jaw joint when opening or closing were evaluated. Any missing permanent tooth expected to be present for that dental age group was recorded as clinically absent tooth.

Intraclass correlation coefficient (ICC) was used to determine intrarater reliability. 30 subjects were reexamined after a period of at least two weeks. Intraclass correlation coefficient (ICC) of 0.94 showed excellent intrarater reliability.

The Statistical Package for Social Sciences, Version 20 (SPSS Inc., Chicago, IL, USA) was used to analyze the data. Chi-square test was used to analyze the data and *P* value was set at *P* < 0.05.

## 3. Results

A total of 684 students (15 to 17 years) from 12 secondary schools in Tehran were examined. 41 students were excluded because they either had received orthodontic treatment or were currently undergoing orthodontic treatment. The final sample comprised 643 subjects (322 males and 321 females).

Table 2 shows the age distribution of the samples. The distribution of grades of treatment need according to the IOTN is shown in Table 3. Orthodontic treatment was required by 9.0 per cent of the population (grades 4-5), and 26.7 per cent were assigned to borderline need (grade 3) and 64.3 per cent to little/no need of orthodontic treatment (grades 1 and 2).  Table 4 shows the prevalence of each occlusal trait in the total sample. The prevalence of Angle Class I malocclusion in this study was higher than other malocclusions (65.2 per cent); Angle Class II and Class III prevalence rates were 24.1 and 10.7 per cent, respectively. 22.7 per cent of the subjects had an overjet greater than 4 mm and 5.9 per cent had a negative overjet. Crowding had the second highest prevalence after Class I malocclusion (62.7%).

## 4. Discussion

In the present study, IOTN was used to record the orthodontic treatment need of the subjects [8]. According to the index, only 10 percent of the total subjects were in severe and extreme need of treatment (IOTN grades 4 and 5). This finding is relatively lower than most European surveys. Chestnutt et al. [10] found that 35% of 12-year-olds and 21% of 15-year-olds across UK had definite need for orthodontic treatment. Perillo et al. [4] reported that 27.3 percent of their sample, which included 703 schoolchildren, was in need of orthodontic treatment. Josefsson et al. [11] compared the frequency of malocclusion and orthodontic treatment need in 12- and 13-year-olds of Swedish and immigrant background and found a high frequency of treatment need in the Swedish group, with 39.5 per cent classified as grades 4 and 5. Puertes-Fernández et al. [12] reported that orthodontic treatment was required by 18.1 per cent of the Saharan population (grades 4-5). In another study conducted to determine the prevalence of malocclusion and orthodontic treatment need in 12- to 16-year-old Spanish schoolchildren, one in every five to six schoolchildren presented an orthodontic treatment need [13]. Nevertheless, they have pointed to the fact that one in four children in their initial sample was receiving or had received orthodontic treatment which might have affected their results. Higher results were also found in nearby regions in the Middle East. Hamdan [14] assess the need for orthodontic treatment among Jordanian schoolchildren and reported that a “definite need” for treatment was recorded in 28% of schoolchildren aged 14 to 17 years.

Nevertheless, the findings of this study are higher than East Asian countries. Esa et al. [15] found that only about 7% of 12-13-year-old schoolchildren in Malaysia had handicapping malocclusion that needed mandatory treatment. However, it must be noted that in the study of Esa et al. [15] Dental Aesthetic Index (DAI) was used to asses perceptions of need for orthodontic treatment. Josefsson et al. [11] reported that the orthodontist's estimate of treatment need by means of IOTN was significantly higher than the subjects' self-assessed need in all of their subjects. Puertes-Fernández et al. [12] also noted that assessment of particular occlusal features by dentists might lead them to overestimate the treatment need.

With regard to the occlusal findings, the highest prevalence was for crowding, which affected more than 62.7% of the subjects. This was similar to the findings of Perillo et al. [4] who also found crowding to have the highest prevalence of occlusal problems which affected 45.9% of their subjects.

## 5. Conclusion

Widespread use of the IOTN along with detailed study of occlusal traits is suitable for planning community dental health resources. In the population of Tehran only 1 in 11 schoolchildren presented an orthodontic treatment need, which is relatively lower than most European countries. However, it should be noted that about 7% of the students had already received orthodontic treatment.

## Figures and Tables

**Table 1:** IOTN treatment grades.

Grade 5 (extreme/need treatment)	
5·i	Impeded eruption of teeth (except for third molars) due to crowding, displacement, the presence of supernumerary teeth, retained deciduous teeth, and any pathological cause
5·h	Extensive hypodontia with restorative implications (more than 1 tooth missing in any quadrant) requiring prerestorative orthodontics
5·a	Increased overjet greater than 9 mm
5·m	Reverse overjet greater than 3.5 mm with reported masticatory and speech difficulties
5·p	Defects of cleft lip and palate and other craniofacial anomalies
5·s	Submerged deciduous teeth

Grade 4 (severe/need treatment)	
4·h	Less extensive hypodontia requiring prerestorative orthodontics or orthodontic space closure to obviate the need for a prosthesis
4·a	Increased overjet greater than 6 mm but less than or equal to 9 mm
4·b	Reverse overjet greater than 3.5 mm with no masticatory or speech difficulties
4·m	Reverse overjet greater than 1 mm but less than 3.5 mm with recorded masticatory and speech difficulties
4·c	Anterior or posterior crossbites with greater than 2 mm discrepancy between retruded contact position and intercuspal position
4·l	Posterior lingual crossbite with no functional occlusal contact in one segment or both buccal segments
4·d	Severe contact point displacements greater than 4 mm
4·e	Extreme lateral or anterior open bites greater than 4 mm
4·f	Increased and complete overbite with gingival or palatal trauma
4·t	Partially erupted teeth, tipped and impacted against adjacent teeth
4·x	Presence of supernumerary teeth

Grade 3 (moderate/borderline need)	
3·a	Increased overjet greater than 3.5 mm but less than or equal to 6 mm with incompetent lips
3·b	Reverse overjet greater than 1 mm but less than or equal to 3.5 mm
3·c	Anterior or posterior crossbites with greater than 1 mm but less than or equal to 2 mm discrepancy between retruded contact position and intercuspal position
3·d	Contact point displacements greater than 2 mm but less than or equal to 4 mm
3·e	Lateral or anterior open bite greater than 2 mm but less than or equal to 4 mm
3·f	Deep overbite complete on gingival or palatal tissues but no trauma

Grade 2 (mild/little need)	
2·a	Increased overjet greater than 3.5 mm but less than or equal to 6 mm with competent lips
2·b	Reverse overjet greater than 0 mm but less than or equal to 1 mm
2·c	Anterior or posterior crossbite with less than or equal to 1 mm discrepancy between retruded contact position and intercuspal position
2·d	Contact point displacements greater than 1 mm but less than or equal to 2 mm
2·e	Anterior or posterior open bite greater than 1 mm but less than or equal to 2 mm
2·f	Increased overbite greater than or equal to 3.5 mm without gingival contact
2·g	Pre- or postnormal occlusions with no other anomalies (including up to half a unit discrepancy)

Grade 1 (no need)	
1·	Extremely minor malocclusions including contact point displacements less than 1 mm

**Table 2:** Age distribution.

Age	Male	Female	Total
15 years old	107 (32.2)	98 (30.5)	205 (31.9)
16 years old	129 (40.1)	114 (35.5)	243 (37.8)
17 years old	86 (26.7)	109 (34)	195 (30.3)

	322	321	643

**Table 3:** Prevalence of the grades of the Dental Health Component of the Index of Orthodontic Treatment Need.

Grade	Female *N* (%)	Male *N* (%)	Total *N* (%)	95% confidence interval
1	93 (29)	65 (20.2)	158 (24.6)	21.27–27.93
2	109 (34)	146 (45.3)	255 (39.7)	35.92–43.48
3	96 (29.9)	76 (23.6)	172 (26.7)	23.28–30.12
4	16 (5)	25 (7.8)	41 (6.4)	4.51–8.29
5	7 (2.1)	10 (3.1)	17 (2.6)	1.37–3.83

Total	321 (100)	322 (100)	643 (100)	

**Table 4:** Prevalence of occlusal variables in the total sample (*N* = 643) (male = 322/female = 321).

Occlusal variables	Male (%)	Female (%)	Total (%)	95% confidence interval
Class I	160 (49.7)	259 (80.7)	419 (65.2)	61.52–68.88
Class I—incisal relationship 1	132	202	334 (51.9)	9.7–14.9
Class I—incisal relationship 2	21	57	78 (12.1)	0.1–1.3
Class I—incisal relationship 3	3	0	3 (0.4)	0.17–1.5
Class I—edge to edge	4	0	4 (0.6)	
Class II	116 (36)	39 (12.1)	155 (24.1)	21.8–27.6
Class II division 1	94	34	128 (19.9)	16.9–23.2
Class II division 2	22	5	27 (4.2)	2.8–6
Class III	46 (14.3)	23 (7.2)	69 (10.7)	8.31–13.09
Class III subdivision	9 (2.8)	33 (10.3)	42 (6.5)	4.7–8.7
Overjet				
Overjet > 4 mm	91 (28.3)	55 (17.1)	146 (22.7)	19.5–26.1
Overjet 0–4 mm	183 (56.8)	248 (77.3)	431 (67)	63.3–70.7
Overjet < 0 mm	31 (9.6)	7 (2.2)	38 (5.9)	4.2–8
Edge to edge	17 (5.3)	11 (3.4)	28 (4.4)	2.9–6.2
Overbite				
Overbite > 4 mm	11 (3.4)	13 (4)	249 (3.7)	2.4–5.5
Overbite 0–4 mm	195 (60.6)	253 (78.8)	448 (69.7)	66–73.2
Overbite < 0 mm	116 (36)	55 (17.1)	171 (26.6)	23.2–30.2
Crossbite				
Unilateral posterior crossbite (left)	26 (8.1)	26 (8.1)	52 (8.1)	6.1–10.5
Unilateral posterior crossbite (right)	21 (6.5)	25 (7.8)	46 (7.1)	5.2–9.4
Bilateral posterior crossbite	29 (9)	19 (5.9)	48 (7.5)	5.6–9.8
None	246 (76.4)	251 (78.2)	497 (77.3)	73.9–80.5
Crowding				
Upper	28 (8.7)	20 (6.2)	48 (7.5)	5.6–9.8
Lower	103 (32)	104 (32.4)	207 (32.2)	28.5–36
Both	72 (22.3)	76 (23.7)	148 (23)	19.8–26.5
None	119 (37)	121 (37.7)	240 (37.3)	33.6–41.2
Lip competency				
Competent	281 (87.3)	261 (81.3)	542 (84.3)	81.5–87.1
Incompetent	41 (12.7)	60 (18.7)	101 (15.7)	12.9–18.6
TMD				
Present (yes)	44 (13.7)	37 (11.5)	81 (13.0)	10.4–15.6
Absent (no)	278 (86.3)	284 (88.5)	562 (87) 87 (87.0)	84.4–89.6
Diastema				
0 mm	272 (84.5)	277 (86.3)	549 (85.4)	82.7–88.1
2 mm	19 (5.9)	28 (8.7)	47 (7.3)	5.3–9.3
3 mm	21 (6.5)	8 (2.5)	29 (4.5)	2.9–6.1
4 mm	5 (1.6)	3 (0.9)	8 (1.2)	0.4–2
5 mm	5 (1.6)	5 (1.6)	10 (1.6)	0.63–2.57

## References

[1] Mohlin B., al-Saadi E., Andrup L., Ekblom K. (2002). Orthodontics in 12-year old children. Demand, treatment motivating factors and treatment decisions. *Swedish Dental Journal*.

[2] Liu Z., McGrath C., Hagg U. (2009). The impact of malocclusion/orthodontic treatment need on the quality of life a systematic review. *Angle Orthodontist*.

[3] van Wyk P. J., Drummond R. J. (2005). Orthodontic status and treatment need of 12-year-old children in South Africa using the dental aesthetic index. *Journal of the South African Dental Association*.

[4] Perillo L., Masucci C., Ferro F., Apicella D., Baccetti T. (2010). Prevalence of orthodontic treatment need in southern Italian schoolchildren. *European Journal of Orthodontics*.

[5] Jamilian A., Toliat M., Etezad S. (2010). Prevalence of malocclusion and index of orthodontic treatment need in children in Tehran. *Oral Health & Preventive Dentistry*.

[6] Thilander B., Pena L., Infante C., Parada S. S., de Mayorga C. (2001). Prevalence of malocclusion and orthodontic treatment need in children and adolescents in Bogota, Colombia. An epidemiological study related to different stages of dental development. *European Journal of Orthodontics*.

[7] Grainger R. M. (1967). Orthodontic treatment priority index. *Vital and Health Statistics 2: Data Evaluation and Methods Research*.

[8] Brook P. H., Shaw W. C. (1989). The development of an index of orthodontic treatment priority. *European Journal of Orthodontics*.

[9] World Health Organization (1985). *Oral Health Care Systems: An International Collaborative Study*.

[10] Chestnutt I. G., Burden D. J., Steele J. G., Pitts N. B., Nuttall N. M., Morris A. J. (2006). The orthodontic condition of children in the United Kingdom, 2003. *British Dental Journal*.

[11] Josefsson E., Bjerklin K., Lindsten R. (2007). Malocclusion frequency in Swedish and immigrant adolescents—influence of origin on orthodontic treatment need. *European Journal of Orthodontics*.

[12] Puertes-Fernández N., Montiel-Company J. M., Almerich-Silla J. M., Manzanera D. (2011). Orthodontic treatment need in a 12-year-old population in the Western Sahara. *European Journal of Orthodontics*.

[13] Manzanera D., Montiel-Company J. M., Almerich-Silla J. M., Gandía J. L. (2009). Orthodontic treatment need in Spanish schoolchildren: an epidemiological study using the Index of Orthodontic Treatment Need. *European Journal of Orthodontics*.

[14] Hamdan A. M. (2001). Orthodontic treatment need in Jordanian school children. *Community Dental Health*.

[15] Esa R., Razak I. A., Allister J. H. (2001). Epidemiology of malocclusion and orthodontic treatment need of 12-13-year-old Malaysian schoolchildren. *Community Dental Health*.

